# Conditional Knockout of PKC-δ in Osteoclasts Favors Bone Mass Accrual in Males Due to Decreased Osteoclast Function

**DOI:** 10.3389/fcell.2020.00450

**Published:** 2020-06-09

**Authors:** Shangfu Li, Tianwei He, Depeng Wu, Liangming Zhang, Ruiqiang Chen, Bin Liu, Jinbo Yuan, Jennifer Tickner, An Qin, Jiake Xu, Limin Rong

**Affiliations:** ^1^Department of Spine Surgery, The Third Affiliated Hospital of Sun Yat-sen University, Guangzhou, China; ^2^Guangdong Provincial Center for Quality Control of Minimally Invasive Spine Surgery, Guangzhou, China; ^3^Guangdong Provincial Center for Engineering and Technology Research of Minimally Invasive Spine Surgery, Guangzhou, China; ^4^School of Biomedical Sciences, The University of Western Australia, Perth, WA, Australia; ^5^Shanghai Key Laboratory of Orthopedic Implants, Department of Orthopedic Surgery, Shanghai Ninth People’s Hospital, Shanghai Jiao Tong University School of Medicine, Shanghai, China

**Keywords:** protein kinase C delta, osteoclast, sex difference, androgen receptor, apoptosis, transcriptome sequencing

## Abstract

Protein kinase C delta (PKC-δ) functions as an important regulator in bone metabolism. However, the precise involvement of PKC-δ in the regulation of osteoclasts remains elusive. We generated an osteoclast specific PKC-δ knockout mouse strain to investigate the function of PKC-δ in osteoclast biology. Bone phenotype was investigated using microcomputed tomography. Osteoclast and osteoblast parameters were assessed using bone histomorphometry, and analysis of osteoclast formation and function with osteoclastogensis and hydroxyapatite resorption assays. The molecular mechanisms by which PKC-δ regulated osteoclast function were dissected by Western Blotting, TUNEL assay, transfection and transcriptome sequencing. We found that ablation of PKC-δ in osteoclasts resulted in an increase in trabecular and cortical bone volume in male mice, however, the bone mass phenotype was not observed in female mice. This was accompanied by decreased osteoclast number and surface, and Cathepsin-K protein levels *in vivo*, as well as decreased osteoclast formation and resorption *in vitro* in a male-specific manner. PKC-δ regulated androgen receptor transcription by binding to its promoter, moreover, PKC-δ conditional knockout did not increase osteoclast apoptosis but increased MAPK signaling and enhanced androgen receptor transcription and expression, finally leding to significant alterations in gene expression and signaling changes related to extracellular matrix proteins specifically in male mice. In conclusion, PKC-δ plays an important role in osteoclast formation and function in a male-specific manner. Our work reveals a previously unknown target for treatment of gender-related bone diseases.

## Introduction

Protein kinase C delta is a member of the highly homologous serine and threonine PKC family, it was the first novel PKC isoform to be identified by screening of mammalian cDNA libraries (Ono et al., 1988). PKC-δ is expressed ubiquitously among cells and tissues, and it is activated in a diacylglycerol and phosphatidylserine dependent but Ca^2+^ independent manner (Steinberg, 2008). Various lines of evidence have shown that PKC-δ, activated in distinct ways, plays a critical role in regulating multiple cellular functions including growth, differentiation, apoptosis, and survival (Roffey et al., 2009).

Bone is maintained by a coupled process called bone remodeling which is characterized by the coordinated activity of bone-forming OBs and bone-resorbing OCs (Iaquinta et al., 2019), and involves a complex series of sequential steps that are highly regulated (Zaidi, 2007). In recent years, extended studies have been carried out to investigate the interrelationship between PKC-δ and skeletal diseases. It is reported that parathyroid hormone activates PKC-δ and regulates osteoblastic differentiation via a PLC-independent pathway (Yang et al., 2006). By using PKC-δ null mice, Cremasco et al. (2012) reported that PKC-δ deficiency perturbs bone homeostasis by selective uncoupling of CTSK secretion and ruffled border formation in OCs via PKC-δ-myristoylated alanine-rich C-kinase substrate (the actin bundling protein) pathway. In addition, further study revealed that global loss of PKC-δ protects against LPS-induced osteolysis owing to an intrinsic defect in osteoclastic bone resorption (Khor et al., 2013). However, the precise involvement of PKC-δ in the regulation of OC biology and bone homeostasis remains elusive.

Here, we generated a mouse strain in which PKC-δ is completely inactivated only in resorbing OCs by breeding mice carrying CTSK promoter-regulated Cre-recombinase with mice possessing loxP recombination sites flanking exon 7 of the PKC-δ gene. Our study aimed to comprehensively characterize the bone phenotype of mice with selective ablation of PKC-δ in OCs; we aimed to dissect the molecular mechanisms by which PKC-δ regulates OC differentiation and function. This study will potentially identify novel molecular targets for OC-associated bone diseases.

## Materials and Methods

### Animal Procedures and Animal Care

All transgenic mice were generated on a C57BL/6J and C57BL/6N genetic background. Mice were group-housed under standard conditions: 12-h light/dark cycle, standard diet containing 1% calcium and 0.76% phosphate, and water *ad libitum* in standard cages. All mice were produced and maintained at the National Resource Center for Mutant Mice Model Animal Research Center of Nanjing University in China according to institutional guidelines. Seven mice per group for both sexes were used for the analysis of bone phenotype using Micro-CT and subsequent histology.

### Generation of Osteoclast-Specific PKC-δ Conditional Knockout Mice

LoxP mice were obtained from RIKEN BioResource Research Center (Stock Number: RBRC06462, Strain Name: C57BL/6-Prkcd<tm1shb>, 3-1-1 Koyadai, Tsukuba, Ibaraki, Japan). CTSK-Cre mice were kindly provided by Professor Jiake Xu from School of Biomedical Sciences, The University of Western Australia. Mice with an OC-cKO of the PKC-δ (PKC-δ cKO) were generated by crossing mice heterozygous for a floxed exon 7 PKC-δ allele (PKC-δ (ex7)^flox/wt^) with CTSK-Cre mice heterozygously carrying a cyclization recombinase of which the expression is controlled by the CTSK promoter (CTSK-Cre^+^PKC-δ^flox/wt^). Offspring were genotyped and the presence of the CTSK-Cre transgene was determined on genomic DNA (gDNA) via PCR with primer sequences presented in Supplementary Table S1. In all the experiments described below, we analyzed CTSK-Cre^+^PKC-δ^flox/flox^ mice that lack PKC-δ in OCs, and CTSK-Cre^–^PKC-δ^flox/flox^ littermates as controls.

### Micro-CT Scanning

Micro structure of bone in mice was measured by high-resolution Micro-CT using a Scanco μCT100 scanner (Brüttisellen, Zurich, Switzerland). Micro-CT analysis was performed on fixed right tibia isolated from euthanized mice scanned with a fixed isotropic voxel size of 10 μm, 100 slices, 70 kV at 200 μA and 300 ms integration time. Standard parameters were then evaluated in the trabecular region of the proximal tibia, commencing at a distance of 0.5 mm from the growth plate and extending a further 1.5 mm distally. Measured parameters included BV/TV (%), trabecular number (Tb.N, mm^–1^), trabecular thickness (Tb.Th, mm) and trabecular separation (Tb.Sp, mm). Cortical bone was analyzed starting at a distance of 2.75 mm from the growth plate and extending 0.5 mm distally to determine total cortical area (Tt.Ar, mm^2^), cortical bone area (Ct.Ar, mm^2^), cortical area fraction (Ct.Ar/Tt.Ar, %) and cortical thickness (Ct.Th, μm).

### Bone Histomorphometry and Immunohistochemistry

Left tibia were fixed overnight in 10% buffered formalin, decalcified with 14% EDTA for 7 days, embedded in paraffin and sectioned (3 μm) for staining. Trabecular bone and *in vivo* OB parameters were analyzed using hematoxylin and eosin (HE) stained sections, while *in vivo* OC parameters were determined from TRAP stained sections as previously described (Yao et al., 2014). Histomorphometric analysis was performed by quantifying parameters including osteoclast surface per bone surface (Oc.S/BS), number of osteoclasts per bone perimeter (N.Oc/B.Pm), osteoblast surface per bone surface (Ob.S/BS) and number of osteoblast per bone perimeter (N.Ob/B.Pm) using an Olympus microscope and the BIOQUANT OSTEO software (BIOQUANT OSTEO 2013 Ver.13.20.6, Nashville, United States). We counted the numbers of positively stained cells in five sequential sections per mouse in each group. Safranin O Fast Green Staining, Massons trichrome staining and Von Kossa staining (in un-decalcified sections) were used to assess chondrocytes, organic and inorganic matrix components in the tibia, respectively. Cartilage thickness was measured in the middle of tibia as previously described (Tong et al., 2019) by using ImageJ software (NIH, Bethesda, MD, United States).

For the un-decalcified bone samples, femoral were dissected, fixed in 70% ethanol, dehydrated and embedded in methyl methacrylate, sagittal sections at 5 μm thickness were undergone von Kossa staining. Trabecular bone volume fraction was analyzed using ImageJ software. For dynamic histomorphometry and bone fluorescent-labeling studies, mice were injected intraperitoneally with calcein (Sigma, 20 mg/kg body weight) and alizarin red complexon (Sigma, 50 mg/kg body weight) at 9 and 2 days, respectively, before sacrifice. The MAR (μm/day) was measured in unstained sections under a fluorescence microscope (Olympus BX-63, Japan) and used to calculate the bone formation rate relative to the bone surface (BFR/BS, μm^3^/μm^2^/year) in the trabecular bone.

For CTSK IHC staining, antigen retrieval was carried out by incubating specimens with bone tissue specific antigen-retrieval solutions (SBT100013, Showbio, Shanghai, China) for 60 min at 37°C. Non-specific binding was blocked with goat serum before incubation with primary antibody against mouse CTSK (ab19027, Abcam, Cambridge, United Kingdom) at 4°C overnight. For detection, sections were treated with HRP-conjugated secondary antibody (GK500505A, Dako, Carpinteria, CA, United States) for 30 min at 37°C, followed by DAB substrate (ZLI-9017, ZSGB-Bio, Beijing, China) for 30 s, counter-stained with Maye’s hematoxylin, dehydrated, and mounted. Semi-quantitative evaluation was performed as previously described (Da et al., 2009) in five random trabecular regions of each section. Two experienced pathologists scored each section which was blinded to them and the final scores were evaluated by consensus.

### Macrophage Isolation From Mouse Bone Marrow, Culture, Osteoclast Differentiation, and TRAP Staining

Bone marrow macrophage from tibia and femur of WT and cKO mice were prepared as previously described (He et al., 2019). BMMs were then seeded on to 96-well plate (6 × 10^3^ cells/well) and cultured in α-MEM supplemented with 30 ng/mL M-CSF (416-ML, R&D system, Minneapolis, MN, United States), and 100 ng/mL RANKL (462-TEC, R&D system). The media was replaced every 2 days and after 7 days of culture the cells were fixed and stained with Acid Phosphatase Staining kit (387A, Sigma-Aldrich, St. Louis, MO, United States) according to the protocol of the manufacturer. TRAP positive multinucleated cells with more than three nuclei were counted as OCs.

### Western Blotting Assay

For short time course signaling pathways, BMMs at 3 × 10^5^ cells/well were seeded into 6-well plates and incubated in complete medium with 30 ng/ml MCSF and 100 ng/ml RANKL for 4 days to allow PKC-δ deletion in OCs. In the next day, cells were starved for 4 h and then 100 ng/ml RANKL were added. Cells were harvested at the time points of 0, 5, 10, 20, 30 and 60 min. For long time periods, BMMs were stimulated with RANKL and MCSF for 0, 1, 3, 5, 7 days. Cells were lysed in RIPA buffer at indicated times for 30 min on ice for protein extraction. An equal amount of proteins (30 μg/lane) were resolved by SDS-polyacrylamide gel electrophoresis and then transferred to a polyvinylidene difluoride membrane (Millipore). The membrane was probed with the indicated primary antibody (in details in Supplementary Table S2), detected using horseradish peroxidase-conjugated secondary antibodies and visualized with ECL reagents (Amersham). α-tubulin was detected on the same membrane and used as a loading control.

### Real-Time RT-PCR Assay

Total RNA from BMMs was extracted with RNAiso Plus (D9108A, Takara, Japan) and reverse transcription was carried out using 1 μg of total RNA with the PrimeScript RT reagent Kit and gDNA Eraser (DRR047A, Takara) in a volume of 20 μl. One microliter of cDNA was amplified with the specific primers (Invitrogen, sequences in Supplementary Table S1) and were quantified on a Light Cycler 480II (Roche) using SYBR green dye (DRR820A, Takara), normalizing with GAPDH. The Ct value of the reference gene GAPDH was subtracted from the Ct value of the target genes (ΔCt), and the average ΔCt value of the triplicates was taken. Relative expression levels of each gene were obtained by using the 2^–ΔΔCt^ method. All the experiments were repeated three times.

### TUNEL Assay

Apoptosis in OCs was identified by using the *In Situ* Cell Death Detection Kit, Fluorescein (11684795910, Roche Diagnostics GmbH, Germany) according to the manufacturer’s instructions. Briefly, after 5 and 7 days of RANKL induction in 35 mm and high glass bottom μ-Dish (81158, Ibidi, Germany), samples were fixed in 4% paraformaldehyde for 1 h at 20°C and permeabilized with 0.1% Triton X-100 solution for 2 min on ice. Then the TUNEL reaction mixtures were added to the samples and incubated for 60 min at 37°C in the dark. Finally the samples were visualized under confocal microscope by using an excitation wavelength of 488 nm and detection in the range of 515–565 nm (green). The actin was detected by phalloidin staining (red).

### Hydroxyapatite Resorption Assay

BMMs (6 × 10^3^ cells/well) were directly seeded into a 96 well Corning Osteo Assay Surface plate (3989, Corning Life Sciences, Tewksbury, MA, United States) to begin the differentiation process. Plates were incubated with a differentiation medium (30 ng/ml M-CSF combined 100 ng/ml RANKL), which was changed every 2 days. After 5 and 7 days, the plates were stripped with 1.2% sodium hypochlorite solution for 5 min to remove cells and air-dried prior to imaging. Overlapping images of the entire well were taken at 20× magnification and these were then used to produce a composite image using Image Composite Editor (ICE 2.0, Microsoft, United States). The total resorption area was measured in the composite image using Image-Pro Plus (version 6.0, Media Cybernetics Company, Rockville, MD, United States).

### Transfection, Transcriptional and Luciferase Assay

Prostate adenocarcinoma cell line (LNCaP, CRL1740) and 293T cell line (CRL3216) were purchased from ATCC. LNCaP cells were maintained in phenol red-free RPMI 1640 supplemented with 10% FBS. 293-T cells were maintained in DMEM supplemented with 10% FBS. Plasmids were obtained from Genomeditech Co., Ltd (Shanghai, China). Cells were transfected with AR firefly luciferase reporter (pGMAR-Lu) plasmid and control renilla luciferase reporter (pGMR-TK) plasmid using Lipofectamine^TM^ 2000 Transfection Reagent (#11668019, Life Technologies) according to the manufacturer’s instruction. Cells were transferred to a 96-well plate 24 h after transfection and were further transfected with PKC-δ plasmid for another 48 h. Finally, luciferase activity was assessed using the Dural-Luciferase^®^ Reporter Assay System (#E1910, Promega, United States) according to the manufacturer’s instructions.

### Transcriptome Sequencing and Bioinformatics Analysis

Total RNA was extracted using RNeasy Mini kit (QIAGEN, Germany) following the manufacturer’s instructions. The quality and integrity of RNA was assessed using the RNA Nano 6000 Assay Kit of the Bioanalyzer 2100 system (Agilent Technologies, CA, United States). A total amount of 3 μg RNA per sample was used for mRNA-Seq library construction using NEBNext Ultra^TM^ RNA Library Prep Kit for Illumina (NEB, United States) according to manufacturer’s recommendations. Heatmap was generated by pheatmap package in R. DEGs analysis was performed using the edgeR R package (3.18.1). The *p*-value was adjusted using the Benjamini and Hochberg method, corrected *p*-value of 0.05 and absolute fold change of 2 was set as the threshold for significantly differential expression. GO enrichment analysis of DEGs was implemented by the clusterProfiler R package, in which gene length bias were corrected, GO terms with corrected *p*-value less than 0.05 were considered significantly enriched by DEGs. KEGG database^1^ was used for KEGG pathway enrichment analysis of DEGs, and clusterProfiler R package was used to test the statistical enrichment of DEGs in KEGG pathways. The RNA-seq data were analyzed by wcgene biotech (shanghai, china).

### Statistical Analysis

All data was pooled from at least three independent experiments. Descriptive statistics included means and standard deviations for continuous variables and percentages for categorical ones. Normality of distribution was assessed by Kolmogorov–Smirnov test. Differences were examined by two tailed Student’s *t*-test for comparing two groups and by one- or two-way analysis of variance (ANOVA) test for comparing multiple groups. When significant differences were indicated by ANOVA, Turkey’s *post hoc* test was used to compare the differences between groups. All data analysis was performed with SPSS 20.0 Package (SPSS software 20.0; SPSS, Chicago, IL, United States). All statistical tests were two-sided and values of *p* smaller than 0.05 were considered significant.

## Results

### Mice With PKC-δ cKO in Osteoclasts Exhibit Increased Bone Mass With Changes in Micro-Structure in a Sex Dependent Manner

Firstly, we confirmed the CTSK-driven PKC-δ deletion in OCs in mice. To analyze a direct effect of PKC-δ deficiency on OCs and bone homeostasis, we used the conditional PKC-δ allele in which exons 7 are flanked by loxP sites. Cre-mediated deletion of exons 7 results in a frame shift and a PKC-δ null allele (Supplementary Figure S1A). For specific deletion in OCs, we crossed the conditional PKC-δ allele to CTSK-Cre mice, efficiency of Cre-mediated deletion of PKC-δ exons 7 and consequent loss of PKC-δ expression in OCs was confirmed by gDNA PCR for the deleted and floxed alleles (Supplementary Figure S1B), together with the significant decrease of PKC-δ mRNA expression (Supplementary Figure S1C, BMMs stimulated with RANKL for 5 days) and protein expression (Supplementary Figure S1D, co-culture with RANKL for 7 days).

**FIGURE 1:**
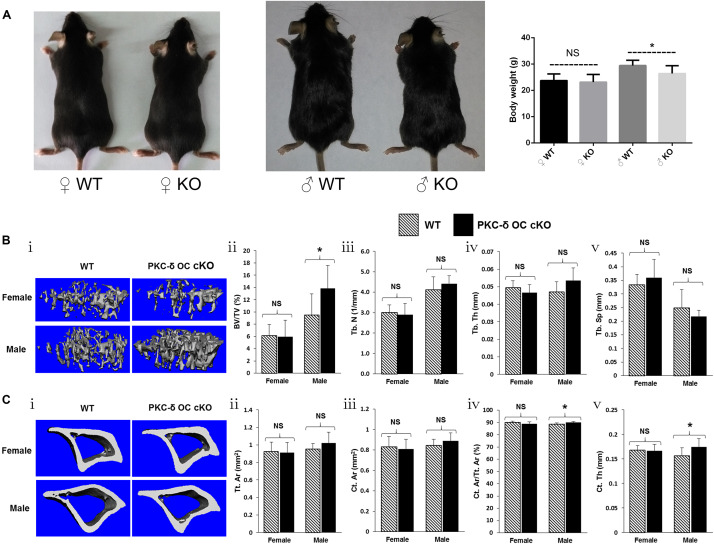
Conditional knockout of PKC-δ in osteoclast resulted in bone mass accrual in 12-week old male mice. **(A)** Representative images and body weight of the pups at 12 weeks of age (WT wild type, KO knockout). **(B,C)** Representative 3D reconstructions of trabecular **(Bi)** and cortical **(Ci)** bone and bone parameters assessed by Micro-CT in proximal tibia in age- and sex-matched WT and PKC-δ conditional knockout mice, respectively (*n* = 7 per group). Trabecular bone parameters **(Bii–**v) are shown as trabecular bone volume fraction (BV/TV, %; **Bii**), trabecular number (Tb.N, 1/mm; **Biii**), trabecular thickness (Tb.Th, mm; **Biv**) and trabecular separation (Tb.Sp, mm; **Bv**). Micro-CT analysis of cortical bone parameters **(Cii–**v) are shown as total cortical area (Tt.Ar, mm^2^; **Cii**), cortical bone area (Ct.Ar, mm^2^; **Ciii**), cortical area fraction (Ct.Ar/Tt.Ar, %; **Civ**) and cortical thickness (Ct.Th, μm; **Cv**). Data are presented as mean/fold change ± SD. NS, non-significant, *p < 0.05 compared with WT control group by two-way ANOVA with Tukey’s *post hoc* test.

To determine the biological consequences of PKC-δ cKO in OCs in skeletal development, we analyzed the bone phenotype of PKC-δ cKO mice *in vivo*. Interestingly, 3-month-old male cKO mice were smaller than WT littermates, in addition, their body weight was lighter (30.5.4 ± 2.2 g vs. 26.8 ± 2.4 g) and statistical difference was found (*p* < 0.05). In contrast, there were no significant changes in female mice regarding their size and body weight (23.5 ± 2.1 g vs. 23.1 ± 2.5 g) (Figure 1A). We further examined the bone micro-structure using Micro-CT and found that male PKC-δ cKO mice exhibited increased trabecular and cortical bone compared to their WT littermates (Figures 1B,C). In the trabecular bone, with statistically significant changes (*p* < 0.05), the percentage of trabecular bone volume versus total volume was about 45% increased in male cKO mice compared to WT (BV/TV, 9.48% vs. 13.87%, Figure 1Bii). Moreover, there were trend of increase in trabecular number (Tb.N, 4.12/mm vs. 4.41/mm, Figure 1Biii) and thickness (Tb.Th, 0.047 mm vs. 0.054 mm, Figure 1Biv) and approximately 10% reduction in trabecular separation (Tb.Sp, 0.25 mm vs. 0.22 mm, Figure 1Bv). However, there were no significant changes in trabecular bone in female cKO mice when compared with the WT controls (Figure 1B). In the cortical bone, statistical differences of increased changes were found in male mice regarding cortical area fraction (Ct.Ar/Tt.Ar, 88.7% vs. 90.0%, Figure 1Civ) and cortical thickness (Ct.Th, 0.156 mm vs. 0.174 mm, Figure 1Cv). While trend of increase were found in total cortical area (Tt.Ar, 0.95 mm^2^ vs. 1.02 mm^2^, Figure 1Cii) and cortical bone area (Ct.Ar, 0.85 mm^2^ vs. 0.89 mm^2^, Figure 1Ciii). As expected, the cortical changes were mild and negligible in cortical bone in female mice (Figure 1C). Taken together, these results revealed an increase in trabecular and cortical bone volume due to ablation of PKC-δ specifically in OCs in a sex dependent maner in male mice.

### Increased Bone Fraction and Decreased Osteoclast in Trabecular Bone of PKC-δ Conditional Knockout Mice

**FIGURE 2:**
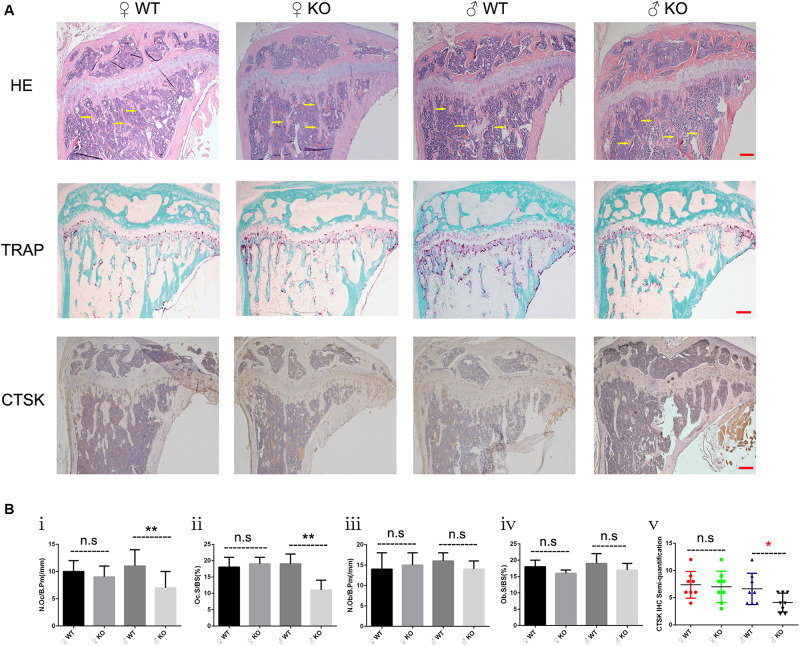
Increased bone fraction and decreased osteoclast number in the trabecular bone of male PKC-δ cKO mice. Analysis of proximal tibia sections of 3-month-old PKC-δ cKO and age-sex matched wild type mice using histology (*n* = 7 per group). **(A)** Representative images of HE/TRAP/CTSK stained tibia sections. Arrows indicate trabecular bone within the tibia. Magnification = 40×, bar represents 200 μm. **(B)** Quantitative histomorphometric analysis of bone parameters: **(Bi)** Number of osteoclasts relative to bone surface (N.Oc/B.Pm, mm^– 1^); **(Bii)** Osteoclast surface relative to bone surface (Oc.S/BS, %); **(Biii)** Number of osteoblasts (N.Ob/B.Pm, mm^– 1^); **(Biv)** Osteoblast surface (Ob.S/BS, %); **(Bv)** Semi-quantification of CTSK IHC staining. Bar charts represent mean ± SD. n.s. no statistical significance, *p < 0.05, **p < 0.01 compared with WT control group by two-way ANOVA with Tukey’s *post hoc* test.

Based on the moderate osteopetrotic phenotype observed by Micro-CT, we further explored the specific *in vivo* cellular changes at the trabecular bone surface of PKC-δ cKO mice. Because bone remodeling is highly coordinated by OC bone resorption and OB bone formation, we determined the effects of PKC-δ deletion in OCs on bone structure, OB and OC parameters. To this end, HE, TRAP and CTSK stained histological sections of tibias from 12 weeks old PKC-δ cKO and WT mice were used for histomorphometric analysis (Figure 2). HE staining displayed increased trabecular bone in PKC-δ cKO male mice (Figure 2A, indicated by yellow arrow), further confirming the Micro-CT findings (Figure 1B). Analysis of TRAP staining revealed a reduction in OC number and surface after PKC-δ ablation specifically in OCs (Figures 2Bi,ii). No significant differences were observed in OB surface and number of OBs in PKC-δ cKO mice when compared to WT littermates (Figures 2Biii,iv). We further verified our finding by CTSK (a specific protein expressed in mature OCs) IHC staining. As expected, semi-quantitative analysis showed decreased CTSK protein levels after inactivation of PKC-δ in OCs (Figure 2Bv).

It was reported that non-cannonical Wnt signaling through G protein-linked PKC-δ activation promoted bone formation (Tu et al., 2007). In addition, studies have shown that PKC-δ is an important regulator of osteochondral plasticity at the interface between articular cartilage and the osteochondral junction using PKC-δ null mice (Yang et al., 2015). To exclude the influence of osteoblast formation and cartilage changes on the bone phenotype after PKC-δ cKO in OCs, we performed bone formation and bone component analysis in the proximal tibia of 12-week-old WT and PKC-δ cKO mice. Representative images of fluorescence double labeling (Supplementary Figures S2Ai–iv) and histomorphometric analysis of the MAR (Supplementary Figure S2Av) and the bone formation rate with a bone surface referent (BFR/BS, Supplementary Figure S2Avi) indicated that PKC-δ cKO did not affect bone formation either in male or female mice. Bone is a dynamic organ composed of organic and inorganic elements, we used von Kossa staining (for inorganic components) and Masson’s trichrome staining (for organic components) to investigate the effect of ablation of PKC-δ specifically in OCs on bone composition. Representative images showed increased inorganic contents in trabecular bone in male mice but not in female mice (Supplementary Figure S2Bi, indicate by yellow arrows) and semi-quantitative analysis revealed increased trabecular bone volume fraction (Supplementary Figure S2Bii). No changes to organic components were observed in either male or female mice (Supplementary Figure S2C). We examined cartilage using Safranin O Fast Green Staining (Supplementary Figure S2Di) and no significant differences of cartilage thickness (Supplementary Figure S2Dii, 126 ± 23 μm vs. 134 ± 18 μm for female and 141 ± 27 μm vs. 145 ± 22 μm for male) were found in both sexes.

Collectively, these data supported the notion that PKC-δ cKO mice exhibited moderate osteopetrosis predominately owing to changes in OC parameters.

### Decreased Osteoclastogenesis and Bone Resorption in Bone Marrow Monocytes From Male PKC-δ cKO Mice

**FIGURE 3:**
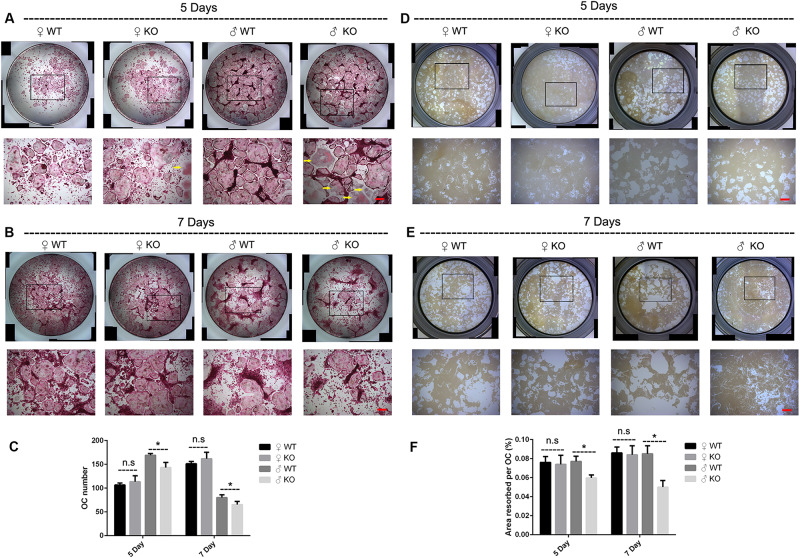
Ablation of PKC-δ specifically in osteoclasts decreased osteoclastogenesis and bone resorption in male mice. **(A,B)** Representative images of osteoclasts with TRAP staining after RANKL (100 ng/ml) induction for 5 days **(A)** and 7 days **(B)**, the square in the upper images of each well indicated where the lower images were captured, the yellow arrow shows unusual morphology of OC in male PKC-δ cKO mice. Magnification = 50×, bar represents 200 μm. **(C)** The number of TRAP positive multinucleated cells (>3 nuclei) per well was quantified. **(D,E)** Representative images of eroded areas after RANKL (100 ng/ml) stimulation for 5 days **(D)** and 7 days **(E)**, the square in the upper images of each well indicated where the lower images were captured. Magnification = 50×, bar represents 200 μm. **(F)** Quantitative analysis of the resorbed proportion per osteoclast by measuring the area of the mineral coating removal. WT wild type, KO knockout. Experiments were carried out in triplicate and results are presented as mean ± SD. Bar represents 200 μm, n.s. no statistical significance, *p < 0.05 vs. WT control group by two-way ANOVA with Tukey’s *post hoc* test.

In addition to the decreased OCs observed in PKC-δ cKO male mice *in vivo*, we further investigated the effects of PKC-δ ablation selectively in OCs on osteoclastogenesis and bone resorption *in vitro*. As shown in Figures 3A–C, BMMs from male PKC-δ cKO produced significantly less OCs than WT littermates after stimulating with RANKL for 5 days (Figures 3A,C) and 7 days (Figures 3B,C), whereas there were no differences in OC number between female mice and WT controls (Figures 3A–C). Interestingly, as indicated by yellow arrows (Figure 3A), we found that male PKC-δ cKO OCs displayed morphology changes (be much more easily to lysis which likes a morphology change of apoptosis). Consistent with OC formation, by using Hydroxyapatite resorption assay, we demonstrated that bone resorption area was also significantly reduced in BMMs of male PKC-δ cKO after incubating with RANKL for both 5 days (Figures 3D,F) and 7 days (Figures 3E,F).

### Enhanced Androgen Receptor Transcription and Expression and Increased MAPK Signaling During Osteoclastogenesis in Male PKC-δ cKO Mice

**FIGURE 4:**
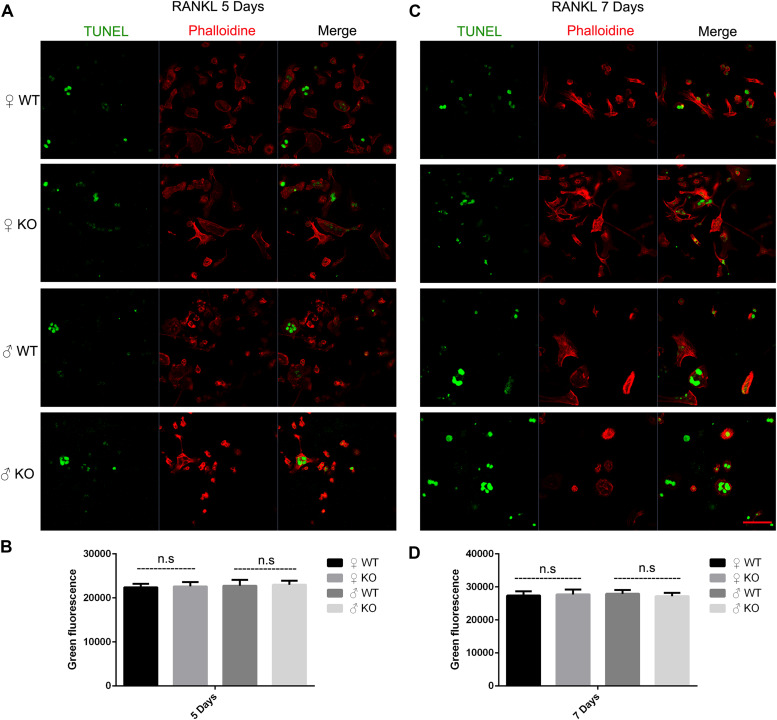
Deletion of PKC-δ selectively in osteoclasts did not increase osteoclast apoptosis during osteoclastogenesis. **(A,C)** Osteoclasts on μ-Dish were immunofluorescently stained with TUNEL and phalloidin to visualize apoptosis and actin by confocal microscopy after RANKL induction for 5 days **(A)** and 7 days **(C)**. Magnification = 400×, bar represents 50 μm. **(B,D)** Semi-quantification of osteoclasts apoptosis was measured in a fluorescence microplate reader after stimulating with RANKL for 5 days **(B)** and 7 days **(D)**. Bar charts represent mean ± SD. Data are representative of three experiments. n.s. no statistical significance compared with WT control group by one-way ANOVA with Tukey’s *post hoc* test.

**FIGURE 5:**
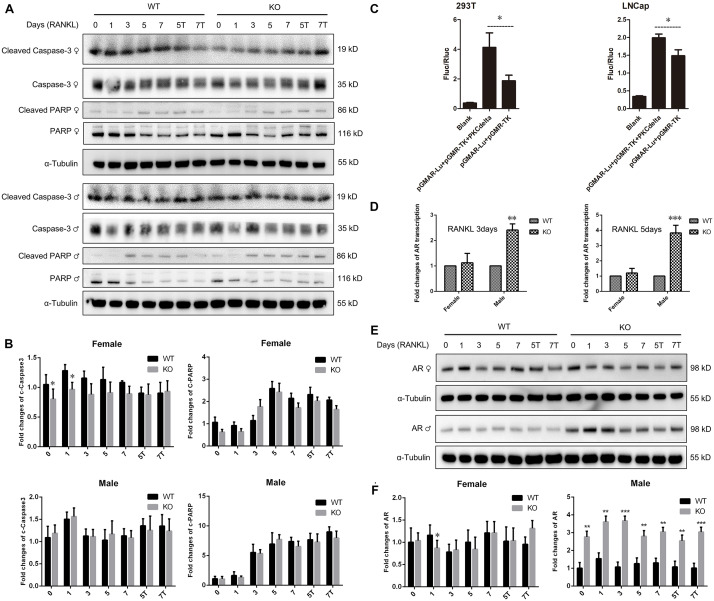
No changes of apoptosis protein expression and significant up-regulation of androgen receptor transcription and expression in male PKC-δ deficient osteoclasts. **(A,B)** Western Blot analysis of characteristic apoptosis proteins (cleaved Caspase-3, Caspase-3, cleaved PARP and PARP) by detecting total protein from WT and PKC-δ cKO BMMs stimulated with RANKL on the indicated days **(A)**; Quantitative analysis of protein expression relative to WT littermate, as measured by densitometry of Western Blot images **(B)**. T 10^– 8^ M testosterone, α-Tubulin was probed as a loading control. Statistical analysis was performed by comparing to WT in each time point for male and female, respectively. **(C)** AR transcriptional activity was significantly enhanced by PKC-δ in 293-T and LNCaP cells by transfection and luciferase assay. pGMAR-Lu AR firefly luciferase reporter, pGMR-TK control renilla luciferase reporter. **(D)** RT-PCR shown that AR transcriptional activity was significantly increased in PKC-δ cKO male mice after stimulating with 100 ng/ml RANKL for 3 days and 5 days, respectively. **(E,F)** Western Blot analysis of AR protein expression by detecting total protein from PKC-δ cKO and WT BMMs stimulated with RANKL on the indicated days **(E)**; Quantitative analysis of AR expression in cKO mice relative to WT littermate by measuring densitometry of Western Blot images **(F)**. T 10^– 8^ M testosterone, α-Tubulin was probed as a loading control. Experiments were repeated three times. *p < 0.05, **p < 0.01, ***p < 0.001 vs. control group by two tailed Student’s *t*-test **(C)** and by two-way ANOVA with Tukey’s *post hoc* test **(B,D,F)**.

Osteoclast apoptosis is associated with cell morphology changes. As we observed obvious morphology changes in OCs during osteoclastogenesis in male PKC-δ cKO mice, we tested whether selective deletion of PKC-δ in OCs affected OC apoptosis. Interestingly, using TUNEL assay, we did not observe significant fluorescence intensity changes during osteoclastogenesis after stimulating with RANKL for 5 days (Figures 4A,B) and 7 days (Figures 4C,D) in either male or female PKC-δ cKO mice. To confirm this finding we further detected protein expression changes characteristic of apoptosis. As shown in Figure 5A, cleaved Caspase-3, Caspase-3, cleaved PARP and PARP protein expression levels in PKC-δ cKO mice were similar to that of WT controls during osteoclastogenesis (Figure 5B), further confirming that ablation of PKC-δ selectively in OC does not promote OC apoptosis.

Because AR is abundantly expressed in male OC and can potently suppress osteoclastogenesis and bone resorption (Huber et al., 2001; Kawano et al., 2003), to further explore the molecular basis for gender dependent changes during OC differentiation and function, we theorized that PKC-δ regulated AR transcription and expression by binding to its promoter. Therefore, we firstly examined the effect of PKC-δ on AR transcriptional activity by transfection and luciferase assay. LNCaP and 293T cells were transfected with AR reporter plasmids. As expected, luciferase activity was dramatically increased after co-transfection with PKC-δ plasmid in both cell lines (Figure 5C), indicating that PKC-δ regulated AR transcription by binding to its promoter. Based on this finding, we further investigated the AR RNA and protein expression changes during osteoclastogenesis in PKC-δ cKO mice (Figures 5D–F). Interestingly, RT-PCR semi-quantification analysis revealed that selective ablation of PKC-δ in OC could greatly enhance AR transcription during osteoclastogenesis both in 3 days and 5 days after RANKL induction only in male mice (Figure 5D). Furthermore, optical density analysis shown that AR protein expression was also significantly elevated at all the time-points (1/3/5/7 days) following RANKL stimulation in male mice but not in female mice (Figures 5E,F).

**FIGURE 6:**
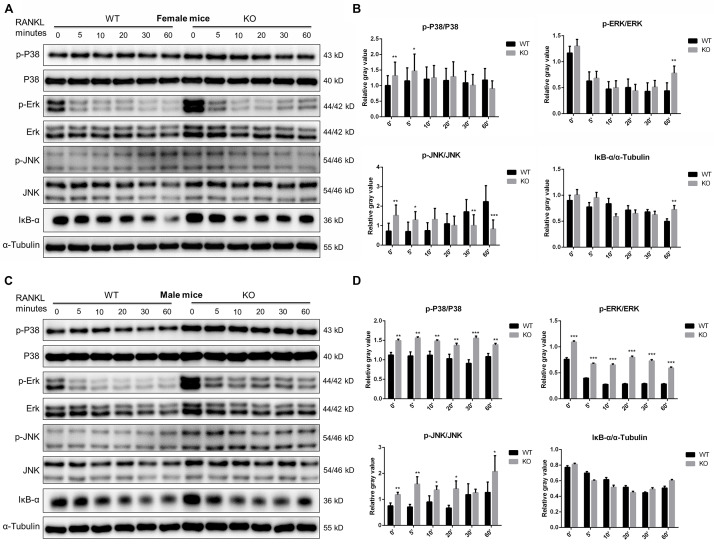
PKC-δ conditional knockout in osteoclasts enhanced RANKL-induced MAPK signaling pathway during osteoclastogenesis in male mice. **(A,C)** Representative Western Blot images of p-JNK, JNK, p-ERK, ERK, p-P38, P-38, IκB-α and α-tubulin at 0, 5, 10, 20, 30, 60 min stimulated by 100 ng/ml RANKL in pre-osteoclasts from PKC-δ cKO and wild type female **(A)** and male **(C)** mice. **(B,D)** The relative ratios of phosphorylated proteins to unphosphorylated proteins or IκB-α to internal control were quantitatively determined in female **(B)** and male **(D)** mice, respectively. The data in the Figures represent the means ± SD. α-Tubulin was used as internal control. Experiments were carried out in triplicate. Significant differences between the cKO and wild type groups are indicated as *p < 0.05, **p < 0.01, ***p < 0.001 by two-way ANOVA with Tukey’s *post hoc* test **(B,D)**.

Since members of the MAPK signaling (including ERK, JNK and P38) and NF-κB signaling pathways play a crucial role in OC survival, differentiation and function (Abu-Amer, 2013; Lee et al., 2018). We further investigated these signaling at 0, 5, 10, 20, 30, 60 min after RANKL stimulation in PKC-δ cKO and wild type mice. As shown in Figure 6, in female mice, although significant changes were found in some time points of p-P38 (0 and 5 min), p-ERK (60 min), and p-JNK (0, 5, 30, and 60 min), the trend of changes was not consistent. Moreover, no significant differences of IκB-α expression were found in cKO mice (Figures 6A,B). By contrast, the phosphorylation of P38 and ERK was remarkably enhanced at all the time points while the phosphorylation of JNK was significantly increased at 0, 5, 10, 20, and 60 min after RANKL treatment in male mice of PKC-δ cKO. However, the expression levels of IκB-α were not changed (Figures 6C,D). These findings suggested that PKC-δ cKO enhanced the RANKL-induced MAKP signaling pathway only in male mice, especially the expression of p-P38 and p-ERK.

Taken together, our data clearly demonstrated that inactivation of PKC-δ specifically in OCs had no effect on OC apoptosis but enhanced AR transcription and expression and increased MAPK signaling during osteoclastogenesis in a sex dependent manner in male mice.

### Conditional Inactivation of PKC-δ in Osteoclasts Led to Significant Gene Expression and Signaling Changes Related to Extracellular Matrix Proteins Only in Male Mice

**FIGURE 7:**
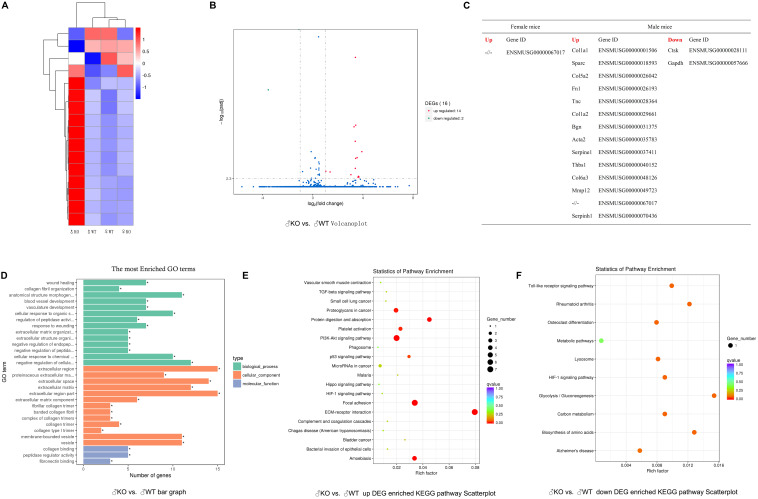
Inactivation of PKC-δ specifically in osteoclasts led to significant alteration of differentially expressed genes (DEGs) and signaling changes only in male mice. **(A)** Heat map of DEGs up and down in 12-week-old PKC-δ cKO mice BMMs derived osteoclasts. **(B)** Volcano plot of data set showing the DEGs in the BMMs stimulating with RANKL for 3 days. Dots with red and green color represented the up-regulated and down-regulated DEGs, respectively, between PKC-δ cKO and WT male mice. **(C)** List of up-regulated and down-regulated DEGs extracted from **(A,B)** with Gene ID and presented in details in Table 1. **(D)** Bar graph of GO enrichment analysis of DEGs showing the most enriched GO terms compared between PKC-δ cKO and WT male mice by two tailed Student’s *t*-test. **(E)** Gene set enrichment analysis of BMMs from KEGG database revealed the most likely up-regulated pathways responsible for the gender differences. **(F)** Scatterplot of KEGG pathway enrichment analysis of DEGs showed the down-regulated pathways comparing PKC-δ cKO and WT male mice. RNA-seq data from three biological replicates per group were used.

To further address the issue of gender differences after PKC-δ cKO, we investigated the gene expression differences in BMMs after stimulating with RANKL by transcriptomics sequencing and bioinformatics analysis. RNA-seq-based expression heat map of PKC-δ cKO mice BMM-derived OCs showed that the lack of PKC-δ altered the expression of many genes much more dramatically in male mice (Figure 7A). We further identified the DEGs responsible for the gender differences by using Volcano plot (Figure 7B): There were 14 up-regulated DEGs and 2 down-regulated DEGs in male mice when compared with WT controls (Figure 7C and Table 1). In contrast, there is only one DEG in female mice (Figure 7C). To reveal the most likely pathways and genes responsible for the sex differences, we performed the GO enrichment analysis (Figure 7D). It is noticeable that most of the DEGs are located in the cellular components of extracellular region and the molecular functions are mainly collagen binding and peptidase regulator activity. We further explored the underlying molecular mechanisms for the gender differences of PKC-δ cKO on OC differentiation and function by KEGG pathway enrichment analysis (Figures 7E,F). The enriched KEGG pathway scatterplot showed that most of the up-regulated DEGs were related to focal adhesion and ECM–receptor interaction (Figure 7E), while the down-regulated DEGs are mainly responsible for OC differentiation and lysosomes (Figure 7F).

**TABLE 1:** Genes differentially expressed during osteoclastogenesis in male PKC-δ conditional knockout mice (*n* = 3).

Gene symbol	Gene name	Fold changes (cKO vs. WT, log_2_)	*p*-value
Col1a1	Collagen type I alpha 1 chain	3.427	1.04E-21
Col1a2	Collagen type I alpha 2 chain	3.389	5.27E-17
Col5a2	Collagen type V alpha 2 chain	3.640	1.70E-06
Col6a3	Collagen type VI alpha 3 chain	3.687	2.73E-06
Sparc	Secreted protein acidic and cysteine rich	3.915	5.38E-14
Fn1	Fibronectin 1	3.404	4.37E-41
Tnc	Tenascin C	3.396	6.15E-09
Bgn	Biglycan	3.045	5.38E-07
Acta2	Actin alpha 2	3.423	6.69E-12
Serpine 1	Serpin family E member 1	3.539	3.45E-12
Thbs1	Thrombospondin 1	3.293	3.59E-21
Mmp12	Matrix metallopeptidase 12	1.042	4.71E-08
Serpinh 1	Serpin family H member 1	3.612	3.40E-06
Ctsk	Cathepsin K	−1.099	0
Gapdh	Glyceraldehyde-3-phosphate dehydrogenase	−3.541	8.60E-32

Collectively, all these results indicated that deletion of PKC-δ specifically in OCs led to alteration of many DEGs and significant signaling changes only in male mice.

## Discussion

In this study, we found that PKC-δ deficiency in OCs favors bone mass accrual in a sex-dependent manner in male mice. Histological analysis revealed increased bone fraction and decreased OCs in the trabecular bone of male mice after cKO. Furthermore, our work suggests that PKC-δ cKO decreased osteoclastogenesis and OC function only in male mice. Finally, deletion of PKC-δ selectively in OCs did not increase OC apoptosis but enhanced AR transcription and expression and increased MAPK signaling in addition to alteration of expression of many genes and signaling changes related to ECM biology during osteoclastogenesis in male mice. Hence, these data clearly showed that ablation of PKC-δ specifically in OCs contibuted to an osteopetrotic phenotype in a sex-dependent fasion in male mice. More importantly, this work revealed the gender differences at both cellular and molecular levels. Our work displayed PKC-δ as an important regulator in OC biology and revealed a previously unknown target for treatment of sex-related bone disease.

The observed effect of loss of PKC-δ resulting in increased trabecular bone is consistent with the previous studies displayed by Cremasco et al. (2012) and Khor et al. (2013). However, there is a discrepancy that the sex differences demonstrated in our study was not observed in their papers on the global KO mice. In addition, the exact role of PKC-δ in OC formation and bone resorption is debatable. It is controversial that both PKC activators (Wang et al., 2003) and inhibitors (Yao et al., 2015) are able to inhibit osteoclastogenesis and bone resorption. PKC-δ was identified as the predominant isoform expressed in OCs among all PKC family (Khor et al., 2013), Khor et al. (2013) found enhanced osteoclastogenesis in PKC-δ null mice, and surmised that this is an attempt to compensate for an intrinsic bone resorption defect in these mice. In contrast, Cremasco et al. (2012) showed that PKC-δ deficiency did not impair OC differentiation in PKC-δ null mice. With respect to the role of PKC-δ on OC function, Cremasco et al. (2012) found that PKC-δ was required for CTSK exocytosis and genetic disruption of PKC-δ profoundly impaired OC bone resorption in bone slices and significantly decreased collagen type I fragment concentration in the culture medium. However, PKC-δ ablation did not impair ruffled border formation or trafficking of lysosomes containing v-ATPase (acidification), leading to no resorption changes in osteologic hydroxyapatite-coated slices in PKC-δ null mice. In contrast, others showed that rottlerin, a PKC-δ specific inhibitor, potently inhibited lysosomal acidification in human OCs (Sorensen et al., 2010). To investigate the exact role of PKC-δ in OC formation and function, we inactivated PKC-δ specifically in OCs by using the Cre-loxp system and found decreased osteoclastogenesis and bone resorption in PKC-δ cKO BMMs in a sex dependent manner in male mice. Consistent with our work, Khor et al. (2013) also showed that inhibition of PKC-δ and knock out of PKC-δ resulted in impaired osteoclastic bone resorption *in vitro*. These conflicting results are indicative that PKC-δ has dual roles in bone remodeling, where it may modulate both bone formation and resorption ultimately influencing the bone turnover process. Because bone is a dynamic organ with complex micro-enviroment, understanding the exact role of PKC-δ in OC biology is critical to dissecting its role in other bone cells, such as osteoblasts, osteocytes, chondrocytes and endothelial cells.

Another contradictory result was that the MAPK signaling was enhanced after PKC-δ ablation selectively in OCs. There may be two reasons for decreased OC differentiation and function accompanied paradoxically with increased MAPK signaling. Firstly, because ERK is responsible for OC survival (Miyazaki et al., 2000) while JNK signaling mediates an anti-apoptotic effect of RANKL in OCs (Ikeda et al., 2008). We assumed that the enhanced survival and anti-apoptotic signaling in OCs may be due to an attempt to compensate for the decreased OC function. Secondly, the MAPK signaling may be not the main signaling after PKC-δ deletion specifically in OCs.

An interesting outcome of our studies is the clear sex differences in bone phenotype and OC differentiation and function after deletion of PKC-δ selectively in OCs. It is an unknown phenomenon and not well described in the hormone-related-gene deficient mice. One example of differences between male and female mice has been found in aromatase-deficient mice, where the female aromatase-ablation mice had an increase in bone turnover resembling early postmenopausal osteoporosis, whereas the male aromatase-deficient mice showed decreases in both osteoblastic and osteoclastic surfaces similar to age-related osteopenia compared with WT littermates (Oz et al., 2000). Another example of a sex difference has been found in the ER knockout mice. where the female mice have decreased bone resorption and increased trabecular bone volume, whereas the bones of the male mice were unaffected by the ablation of the ER gene (Sims et al., 2002). However, we have identified two potential explanations that may account for the observed sex differences in PKC-δ cKO mice. Firstly, the presence of functional ARs in OCs (Almeida et al., 2017) indicates that OCs are able to respond directly to androgen through the AR (Pederson et al., 1999), resulting in a suppressive effect on bone resorption (Kawano et al., 2003). The sex-specific effects of androgen on gene expression in human monocyte-derived OCs indicate that AR function is indispensable for male bone formation and remodeling (Wang and Stern, 2011). Moreover, androgens regulated PKC-δ transcription and modulated its apoptotic function in prostate cancer cells (Gavrielides et al., 2006), and prenatal testosterone exposure induces hypertension in adult females via an AR-dependent PKC-δ-mediated mechanism (Blesson et al., 2015). These studies and our results suggest that PKC-δ has a direct interaction with the AR promoter. The studies above lead us to speculate that deletion of PKC-δ in OCs may suppress OC function through increasing AR expression. By using qPCR-based array, Wang and Stern (2011) showed that although OCs from both male and female mice responded to 17β-estradiol and testosterone, the effects of both estrogen and androgen differ in the two sexes, highlighting the importance of considering gender in the design of therapeutics. This study further supports our findings of sex specific effects of PKC-δ. Secondly, transcriptome sequencing and bioinformatics analysis showed that inactivation of PKC-δ specifically in OCs led to up-regulation of only one pseudogene without function in female mice. In contrast, many genes were altered (Colla1, Colla2, Col5a2, Col6a3, Thbs1, Bgn, Tnc, Sparc, Fn1, Acta2, Serpinel, Mmp12, Serpinh1 were up-regulated while Ctsk and Gapdh were down-regulated) during osteoclastogenesis after PKC-δ ablation in male mice. It should be noted that these DEGs are mainly responsible for ECM and collagen synthesis, degradation and signaling [for example Thbs1 (Amend et al., 2015), Bgn (Shirakura et al., 2017), Sparc (Rosset and Bradshaw, 2016), Fn1 (Magnan et al., 2018), and Serpinh1 (Mimura et al., 2016)]. Interestingly, most of these DEGs play important roles in OC biology (especially Col6a3 (Mullin et al., 2018), Bgn (Bi et al., 2006; Kram et al., 2017), Thbs1 (Kukreja et al., 2009; Amend et al., 2015; Koduru et al., 2018), Tnc (Baniwal et al., 2012), Sparc (Ma et al., 2017), and Acta2 (Mullin et al., 2014)] and also displayed sexual dimorphism in regulating many cellular processes. With respect to collagen related genes, postnatal changes and sexual dimorphism were found in collagen expression in mouse skin. Col1a1 and Col1a2 mRNAs increased noticeably at day 30 and remained at high levels until day 120 in male mice, Col3a1mRNA also showed significantly high levels at day 120 in male mice as compared to female. Moreover, testosterone and its effect on collagen expression are responsible for the skin sexual dimorphism (Arai et al., 2017). Intriguingly, the Col5a1 gene is associated with increased risk of anterior cruciate ligament ruptures only in female participants (Posthumus et al., 2009). Bgn-deficient mice are resistant to OVX-induced trabecular bone loss and there is a gender difference in response to Bgn deficiency (Nielsen et al., 2003). For Fn1 and Tnc, tendon of the female mice had approximately twofold elevations in ECM proteins such as Fn1 and Tnc compared with male mice (Sarver et al., 2017). For Acta2, male MDR2 knockout mice tended to have a more pronounced reversal of liver fibrosis than females treated with corticosterone (Petrescu et al., 2017). For Mmp9, less elastin in the aneurysm wall in women than that in men, and the simultaneous higher level of Mmp-9, suggesting differences in the elastolytic process in abdominal aortic aneurysms between the sexes (Villard et al., 2014); in addition, male mucopolysaccharidosis type I mice have an increased incidence of aortic insufficiency associated with an increase in MMP-12 aortic arch content (Tolar et al., 2009). For CTSK, sexual dimorphism existed in MAPK-Activated protein kinase-2 regulation of RANKL-induced osteoclastogenesis in OC progenitor subpopulations (Herbert et al., 2015). Collectively, all these DEGs are closely related to the ECM and the majority display sexual dimorphism. It is reasonable to assume that the gender difference found in the studies above also could be found in other deficient mice; herein it is important to examine the phenotype of both sexes. Although the precise role of PKC-δ in ECM-related gene signaling is complex and remains to be elucidated, it is possible that PKC-δ may regulate AR activity through the DEGs above. However, the underlying molecular mechanisms of the differential expression and the role of these genes in sex related differences in OC functions need to be further investigated.

Limitations of our study should be acknowledged. First, it is reported that CTSK-Cre caused unexpected germline deletion of genes in mice which was found to be due to the expression of CTSK in both testis and ovary (Winkeler et al., 2012). This illustrates that we need to be alert to potential germline recombination which may confound our results regarding the changes of size and androgen signaling. It is reported that lack of PKC-δ (PKC-δ null mice) disrupts fertilization and embryonic development in both males and females (Ma et al., 2015). Testis and ovary dysfunction and hypogonadism are always accompanied with osteopenia (Watts et al., 2012; Karakas and Surampudi, 2018). However, in our study, we found that PKC-δ deficiency in OCs favored bone mass accrual and enhanced AR transcription and expression during osteoclastogenesis only in male mice, furthermore, the reproduction function was not affected after PKC-δ cKO in OCs (unpublished data), suggesting that the proposed effects resulting from germline deletion of PKC-δ are contradictory to our results. In addition, CTSK is mainly expressed in OCs, the expression levels of CTSK in other tissues are much lower than that in bone (Soderstrom et al., 1999). Taken together, we speculate that the CTSK expression in gonads may not confound our results. Although many of the OC-targeted Cre-deleter strains, including CTSK, TRAP and *LysM*, are imperfect and each model has its own limitations (Dallas et al., 2018), the CTSK-Cre mouse is the most widely used Cre transgenic mouse line for the *in vivo* study of OCs, their careful use will continue to provide key advances in our understanding of OC function in bone health and disease. Second, it is difficult to transfect pre-OC of the RAW264.7 cell line and BMMs with plasmids, hence the lack of evidence showing direct interaction between PKC-δ and AR in OC. However, many lines of evidence from other cell lines, such as mesenteric artery smooth muscle cells (Blesson et al., 2015), 293-T and LNCaP cells, support our findings. Clinical and genetic studies of PKC-δ gene mutations in humans are important to confirm these pre-clinical studies in mice, blood and bone samples from patients with PKC-δ deficiency will provide further information to address this issue (Belot et al., 2013; Kuehn et al., 2013; Salzer et al., 2013; Kiykim et al., 2015).

In summary, our data clearly demonstrate sex differences in the function of PKC-δ in mice. More importantly, we provide compelling evidence demonstrating that the sex differences are closely related to AR, MAPK, and ECM related signaling, unveiling an important role for PKC-δ in the pathogenesis of osteoporosis in animal models, cell culture, and molecular interactions. This work contributes to our understanding of the role of PKC-δ in OC bone resorption and might aid in the discovery of novel therapeutic targets for treatment of gender-related bone disorders.

## Data Availability Statement

The raw data supporting the conclusions of this article will be made available by the authors, without undue reservation, to any qualified researcher. The metagenomic sequencing data have been deposited into Sequence Read Archive (SRA) under accession no. PRJNA555653.

## Ethics Statement

All the experiments were approved by the Institutional Animal Care and Use Committee of the Third Affiliated Hospital of Sun Yat-sen University (Approval No: IACUC-F3-18-1202) and were performed according to EU Directive 2010/63/EU.

## Author Contributions

SL and LR conceived the ideas. SL, JX, and AQ planned experiments. SL, TH, DW, and JY performed experiments and analyzed the data. JX and AQ contributed reagents. SL wrote the manuscript. JT and JX revised manuscript content. All authors reviewed the manuscript.

## Disclosure

The funding sponsors had no role in the design of the study; in the collection, analyses, or interpretation of data; in the writing of the manuscript, and in the decision to publish the results. No benefits in any form have been or will be received from a commercial party related directly or indirectly to the subject of this article.

## Conflict of Interest

The authors declare that the research was conducted in the absence of any commercial or financial relationships that could be construed as a potential conflict of interest.

## Footnotes

1 http://www.genome.jp/kegg/

## Abbreviations

Acta2: actin alpha 2
AR: androgen receptor
α-MEM: alpha modified Minimal Essential Medium
Bgn: biglycan
BMM: bone marrow macrophage
BV/TV: bone volume/total volume
cKO: conditional knockout
Col1a1: collagen type I alpha 1 chain
Col1a2: collagen type I alpha 2 chain
Col5a2: collagen type V alpha 2 chain
Col6a3: collagen type VI alpha 3 chain
Ct.Ar: cortical bone area
Ct.Ar/Tt.Ar: cortical area fraction
CTSK: cathepsin K
Ct.Th: cortical thickness
DMEM: Dulbecco’s modified Eagle’s medium
DEG: differentially expressed gene
ECM: extracellular matrix
ER: estrogen receptor
ERK: extracellular signal-regulated kinase
Fn1: fibronectin 1
GAPDH: glyceraldehyde-3-phosphate dehydrogenase
GO: Gene Ontology
IHC: Immunohistochemistry
JNK: c-Jun amino-terminal kinase
LNCaP: lymph node carcinoma of the prostate
MAPK: mitogen-activated protein kinase
MAR: mineral apposition rate
M-CSF: macrophage colony stimulating factor
Micro-CT: microcomputed tomography
Mmp12: matrix metallopeptidase 12
N.Ob/B.Pm: number of osteoblast per bone perimeter
N.Oc/B.Pm: number of osteoclasts per bone perimeter
OB: osteoblast
Ob.S/BS: osteoblast surface per bone surface
OC: osteoclast
Oc.S/BS: osteoclast surface per bone surface
PKC- δ: protein kinase C delta
RANKL: receptor activator of NF- κ B ligand
Serpine 1: serpin family E member 1
Serpinh 1: serpin family H member 1
Sparc: secreted protein acidic and cysteine rich
Tb.N: trabecular number
Tb.Sp: trabecular separation
Tb.Th: trabecular thickness
Thbs1: thrombospondin 1
Tnc: tenascin C
TRAP: tartrate-resistant acid phosphatase
Tt.Ar: total cortical area.
