# Supplementary material for: Conditional Knockout of PKC-δ in Osteoclasts Favors Bone Mass Accrual in Males Due to Decreased Osteoclast Function
Source: Front Cell Dev Biol. 2020 Jun 9;8:450. doi: 10.3389/fcell.2020.00450 (PMC7295979; doi:10.3389/fcell.2020.00450)
Supplement: Supplementary file 1 [file Presentation_1.pdf]

Supplementary Material

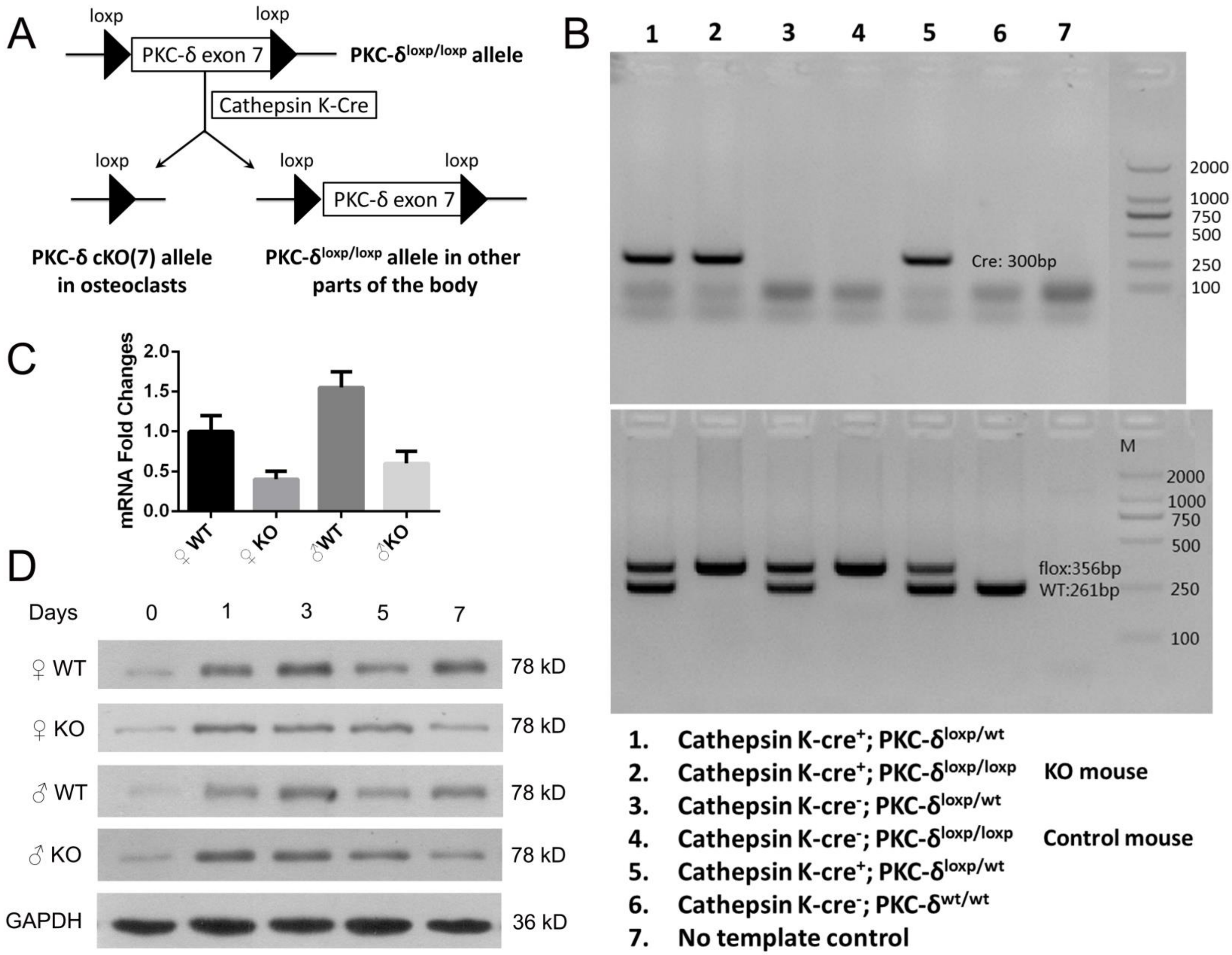

Supplementary Fig. 1 Generation and confirmation of Cathepsin-K-driven PKC-δ deletion in osteoclasts in mice. (A) Strategy for inserting flox sites into the mouse PKC-δ gene; (B) PCR analysis of extracted gDNA from tails derived from wide type, PKC-δ cKO and flox control mice. The 300 bp PCR fragment is detected in PKC-δ cKO mice, indicating Cre-mediated excision; (C) Quantitative RT-PCR analysis of PKC-δ mRNA and validation of PKC-δ-targeted mouse osteoclast cKO in BMMs stimulated with RANKL for 5 days of PKC-δ cKO and wide type control mice. Values are expressed as the mean of three independent experiments ± SD; (D) Western Blot analysis of PKC-δ in BMMs co-culture with RANKL in the indicated days in PKC-δ cKO and wide type control mice, validating efficacy of PKC-δ ablation for floxed allele compared to PKC-δ germline wild-type control.

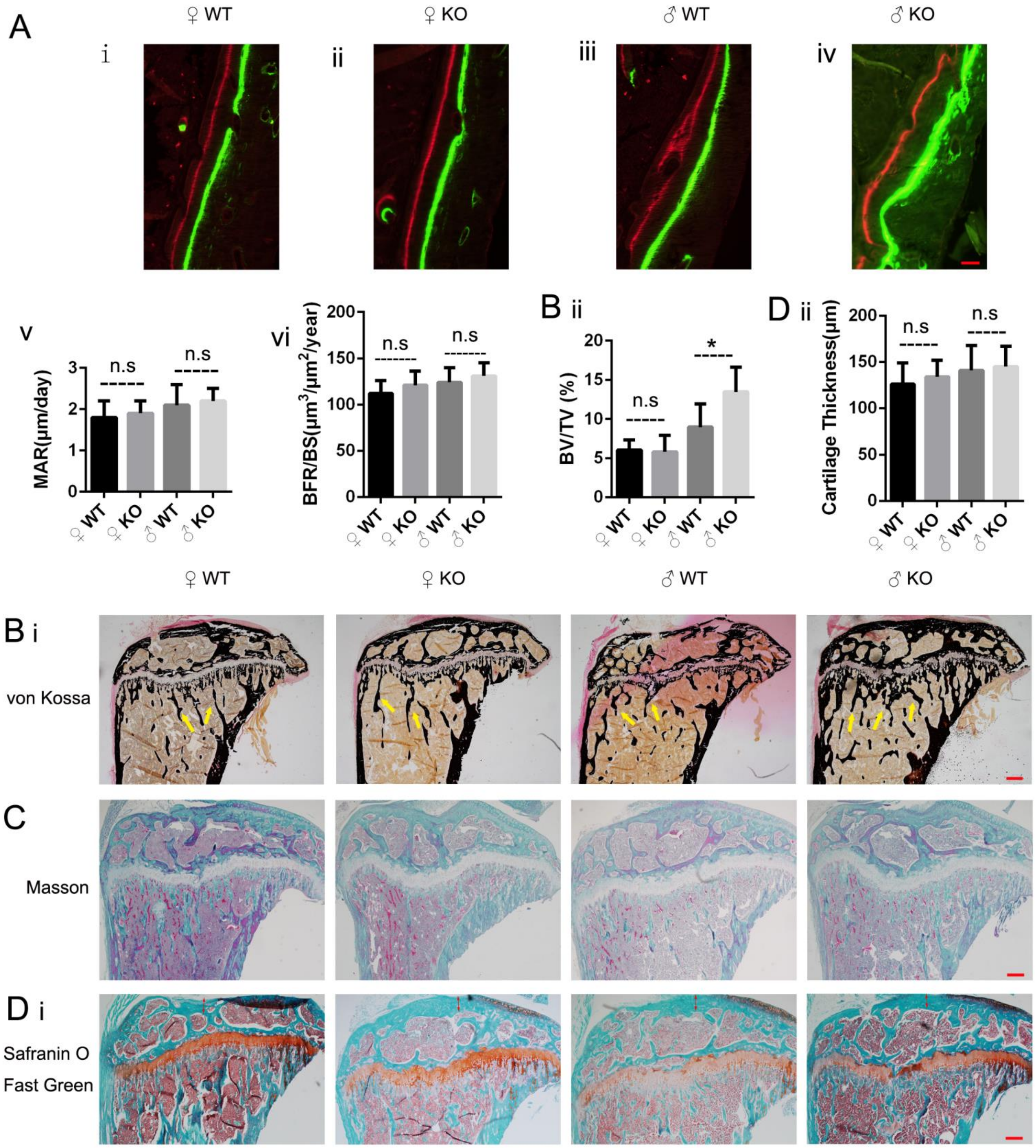

Supplementary Fig. 2 Bone formation analysis and representative images of bone component analysis from proximal tibia of 12-week-old WT and PKC-δ cKO mice. (A) Representative images of fluorescence double labeling (i-iv) to visualize bone formation and histomorphometric analysis of the mineral apposition rate (MAR, v) and the bone formation rate relative to the bone surface (BFR/BS, vi). n.s. no statistical significance compared with WT control group; (B) Representative images of von

Kossa staining to determine changes of inorganic elements in bone. Arrows indicate the trabecular bone (i) and analysis of trabecular bone volume fraction (BV/TV, %; ii). n.s. no statistical significance, *$p<0.05$ vs. WT control group; (C) Representative images of Masson staining to visualize changes of organic elements in bone; (D) Representative images of safranin O staining to determine articular cartilage and double head red arrows depict the thickness of the articular cartilage (i). Quantitative analysis of cartilage thickness was shown in Dii. Bars represent 50μm for A and 200μm for B, C and D respectively.

Supplementary Table 1 Primer sequences used in gDNA PCR and qRT-PCR

Genes	Forward (5'-3')	Reverse (5'-3')	Purpose
PKC-δ	GCCAGCCTTTCTGTGCTGTG	TCCACTCAGGATACATGGTGGG	gDNA PCR
PKC-δ	CAGACCAAGGACCACCTGTT	CGTCCCTGTCTAGCATCACA	qRT-PCR
AR	TGGTAGCTGGTACTTCTAATGC-3	CATAAGGTCCGGAGTAGTTCTC	qRT-PCR
GAPDH	ACCCAGAAGACTGTGGATGG	CACATTGGGGGTAGGAACAC	qRT-PCR

Supplementary Table 2 Antibodies used in Western blotting assay

Names	Company	Catalog Number
Caspase-3	Cell Signaling Technologies	#9665
cleaved Caspase-3	Cell Signaling Technologies	#9664
PARP	Cell Signaling Technologies	#9542
cleaved PARP	Cell Signaling Technologies	#9548
extracellular signal-regulated kinase (ERK)	Cell Signaling Technologies	#4695
p-ERK	Cell Signaling Technologies	#4370
c-Jun amino-terminal kinase (JNK)	Cell Signaling Technologies	#9252
p-JNK	Cell Signaling Technologies	#4668
P-38	Cell Signaling Technologies	#8690
p-P38	Cell Signaling Technologies	#4511
androgen receptor (AR)	Abcam	ab133273
PKC-δ	Abcam	ab182126

IκB-α	Abcam	ab7217
CTSK	SANTA CRUZ	sc-48353
